# Antinociceptive Effect of 3-(2,3-Dimethoxyphenyl)-1-(5-methylfuran-2-yl)prop-2-en-1-one in Mice Models of Induced Nociception

**DOI:** 10.3390/molecules21081077

**Published:** 2016-08-22

**Authors:** Nur Izzati Ismail, Lee Ming-Tatt, Nordin Lajis, Muhammad Nadeem Akhtar, Ahmad Akira, Enoch Kumar Perimal, Daud Ahmad Israf, Mohd Roslan Sulaiman

**Affiliations:** 1 Department of Biomedical Sciences, Faculty of Medicine and Health Sciences, Universiti Putra Malaysia, 43400 Serdang, Malaysia; nurizzati@cyberauthmed.edu.my (N.I.I.); ahmadakira@upm.edu.my (A.A.); enoch@upm.edu.my (E.K.P.); daud.israf@gmail.com (D.A.I.); 2 Faculty of Pharmaceutical Sciences, UCSI University, 56000 Cheras, Malaysia; mingtatt7286@gmail.com; 3 Laboratory of Natural Products, Institute of Bioscience, Universiti Putra Malaysia, 43400 Serdang, Malaysia; nordinlajis@gmail.com; 4 Faculty of Industrial Sciences & Technology, University Malaysia Pahang, 26300 Gambang, Malaysia; nadeemupm@gmail.com

**Keywords:** 3-(2,3-dimethoxyphenyl)-1-(5-methylfuran-2-yl)prop-2-en-1-one, chalcone, antinociceptive activity, opioidergic system, TRPV1 receptor, glutamatergic system

## Abstract

The antinociceptive effects produced by intraperitoneal administration of a novel synthetic chalcone, 3-(2,3-dimethoxyphenyl)-1-(5-methylfuran-2-yl)prop-2-en-1-one (DMFP), were investigated in several mouse models of induced nociception. The administration of DMFP (0.1, 0.5, 1.0 and 5.0 mg/kg) produced significant attenuation on the acetic acid-induced abdominal-writhing test. It also produced a significant increase in response latency time in the hot-plate test and a marked reduction in time spent licking the injected paw in both phases of the formalin-induced paw-licking test. In addition, it was also demonstrated that DMFP exhibited significant inhibition of the neurogenic nociceptive response induced by intraplantar injections of capsaicin and glutamate. Moreover, the antinociceptive effect of DMFP in the acetic acid-induced abdominal-writhing test and the hot-plate test was not antagonized by pretreatment with a non-selective opioid receptor antagonist, naloxone. Finally, DMFP did not show any toxic effects and/or mortality in a study of acute toxicity and did not interfere with motor coordination during the Rota-rod test. Our present results show that DMFP exhibits both peripheral and central antinociceptive effects. It was suggested that its peripheral antinociceptive activity is associated with attenuated production and/or release of NO and various pro-inflammatory mediators, while central antinociceptive activity seems to be unrelated to the opioidergic system, but could involve, at least in part, an interaction with the inhibition of capsaicin-sensitive fibers and the glutamatergic system.

## 1. Introduction

Pain remains a major issue in a vast array of medical conditions. Although many effective and potent pharmacotherapies such as opioids and non-steroidal anti-inflammatory analgesic drugs are available, they all have limitations. The continuous use of these analgesic drugs is generally followed by undesirable adverse side effects, such as gastrointestinal damage, renal toxicity, tolerance and respiratory depression [1,2,3]. Therefore, the search for new analgesic compounds with good efficacy and least undesirable side effects to alleviate this obstinate painful condition is potentially very important and is now an interesting strategy. Taking these into consideration, compounds showing an analgesic effect have appeared as attractive therapeutic sources for the development of new relevant drugs for the management of several painful conditions.

Chalcones (1,3-diaryl-2-propen-1-ones) represent an important group of natural or synthetic compounds belonging to the flavonoid family. They have been reported to exhibit broad spectrum of biological and pharmacological activities, such as antimicrobial [4], anticancer [5], antiulcer [6], antinociceptive [7,8,9], anti-inflammatory [10], among others, and thus comprise a class with important therapeutic potential. Many chalcones and their synthetic derivative analogues have been found to inhibit the synthesis of nitric oxide (NO) and prostaglandins (PG), which are products of the nitric oxide synthase (NOS) and cyclooxygenase (COX) pathways, respectively. These pathways constitute the major proinflammatory pathways and remain the most targeted for anti-inflammatory and antinociceptive drugs development [11,12,13,14].

**Figure 1:**
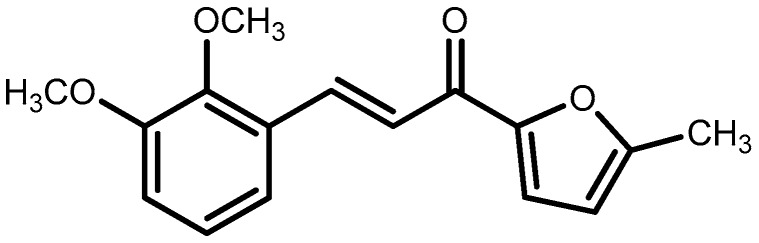
Chemical structure of 3-(2,3-dimethoxyphenyl)-1-(5-methylfuran-2-yl)prop-2-en-1-one (DMFP).

Our ongoing interest in the search for novel analgesic agents has led to the investigation of naturally occurring chemical resources [15,16,17,18] as well as synthetic analogues based on natural products [9,19]. Recently, we have investigated that chalcones bearing substituted furanyl group, 3-(2,3-dimethoxyphenyl)-1-(5-methylfuran-2-yl)prop-2-en-1-one (DMFP, Figure 1) showed remarkable anti-inflammatory activity. It was demonstrated that this compound significantly and potently suppressed NO in interferon-c (IFN-c)- and lipopolysaccharide (LPS)-activated RAW 264.7 cells [20]. In light of the significant activity of DMFP in the attenuation of NO-related effects, which have also been associated with the development of pain, this compound was further synthesized in our laboratory and evaluated with respect to its antinociceptive properties. We herein report the findings on the antinociceptive activity of DMFP using several in vivo experimental models of chemicals- and heat-induced nociception in mice and elucidate some of its possible mechanisms of action.

## 2. Results

### 2.1. Antinociceptive Studies

#### 2.1.1. Acetic Acid-Induced Writhing Test

**Figure 2:**
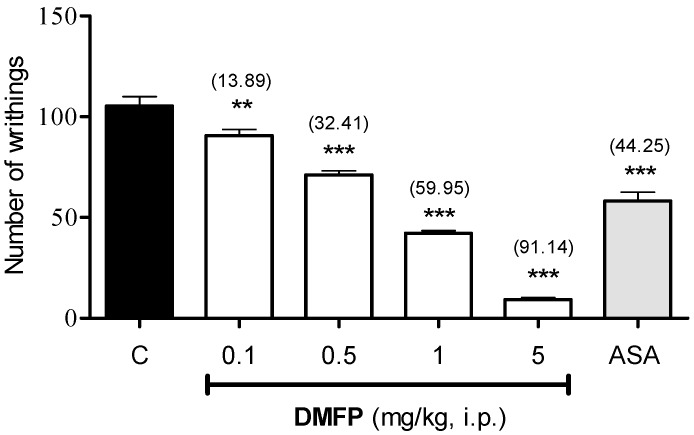
Effect of DMFP (0.1, 0.5, 1 and 5 mg/kg, i.p.) in acetic acid-induced abdominal-writhing test in mice. Each column represents the mean ± S.E.M. (*n* = 6). The mice were pretreated with vehicle (C, 10 mL/kg, i.p.), DMFP (0.1, 0.5, 1.0 and 5.0 mg/kg, i.p.) or acetylsalicylic acid (ASA, 100 mg/kg, i.p.). The asterisks denote significance levels ** *p* < 0.01, *** *p* < 0.001, when compared with the control vehicle group. Statistical significance was determined by one-way ANOVA followed by Dunnett’s *post-hoc* test. Values in parentheses are percentages of inhibition.

The effect of DMFP on writhing response in mice is shown in Figure 2, DMFP (0.1, 0.5, 1.0, and 5.0 mg/kg, i.p.) administered intraperitoneally (i.p.) caused dose-dependent inhibition on the writhing response induced by acetic acid with 13.89% (*p* < 0.01), 32.41% (*p* < 0.001), 59.95% (*p* < 0.001) and 91.14% (*p* < 0.001) of inhibition as compared to control, respectively. Such effect was also observed in mice pre-treated by ASA with 44.25% (*p* < 0.05) inhibition. The calculated mean ID_50_ for i.p. administered DMFP in this model was 0.85 mg/kg (CI, 0.76 to 0.95 mg/kg).

#### 2.1.2. Formalin-Induced Paw-Licking Test

**Figure 3:**
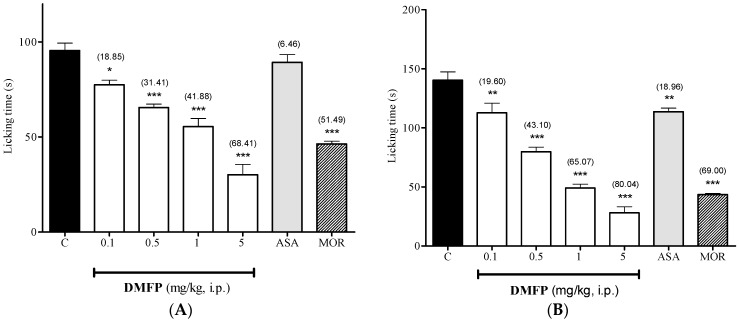
Effect of DMFP in formalin-induced paw-licking test (early phase, panel (**A**); and late phase, panel (**B**) in mice. Each column represents the mean ± S.E.M. (*n* = 6). The mice were pretreated with vehicle C, 10 mL/kg, i.p.), DMFP (0.1, 0.5, 1.0 and 5.0 mg/kg, i.p.), acetylsalicylic acid (ASA, 100 mg/kg, i.p.) or morphine (MOR, 5 mg/kg, i.p.). The asterisks denote the significance levels * *p* < 0.5, ** *p* < 0.01, *** *p* < 0.001, when compared with control vehicle group. Statistical significance was determined by one-way ANOVA followed by Dunnett’s *post-hoc* test. Values in parentheses are percentage of inhibition.

As shown in Figure 3, the i.p. treatment with DMFP at the doses of 0.1, 0.5,1.0 and 5.0 mg/kg significantly inhibited the licking time in of both neurogenic (0–5 min, panel A), by 18.85% (*p* < 0.5), 31.41% (*p* < 0.001), 41.88% (*p* < 0.001) and 68.41% (*p* < 0.001), and inflammatory (15-30 min, panel B), by 19.60% (*p* < 0.01), 43.10% (*p* < 0.001), 65.07% (*p* < 0.001) and 80.04% (*p* < 0.001), phases of formalin-induced paw-licking test, when compared to control group. The treatment with ASA (100 mg/kg, i.p.) only significantly inhibited the licking time in inflammatory phase by 18.96% (*p* < 0.01). On contrary, morphine (5 mg/kg, i.p.) significantly inhibited both phases of the test by 51.49% and 69.00% (*p* < 0.001), respectively.

#### 2.1.3. Hot-Plate Test

**Figure 4:**
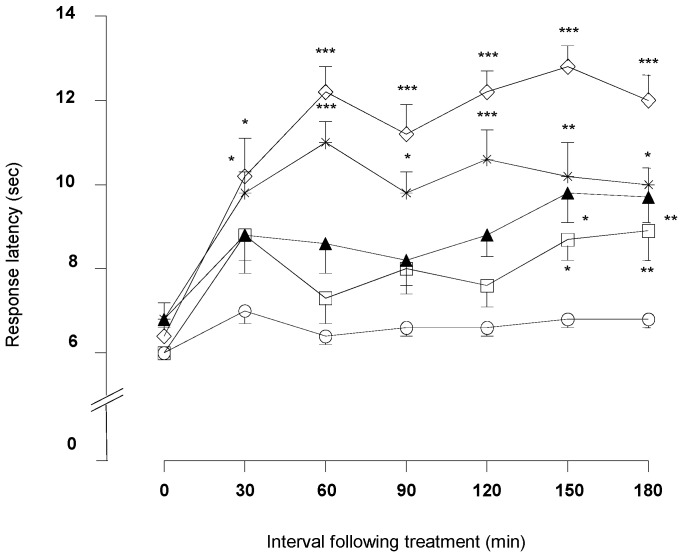
Effect of DMFP on the hot plate test in mice. Results are expressed in mean ± S.E.M. of response latency (s) of 6 mice. Statistical significance was denoted by one-way ANOVA followed by Dunnett’s *post-hoc* test. * *p* < 0.05; ** *p* < 0.01; *** *p* < 0.001 compared with control group; Control (○), DMFP (□, 0.1; ▲, 0.5; ✳ 1.0; ◇ 5.0 mg/kg, i.p.).

As depicted in Figure 4, DMFP treatment at doses of 1.0 and 5.0 mg/kg significantly increased the response latency to a heat stimulus 30 min after the treatment and persisted until 180 min in comparison to the control group (vehicle 10 mL/kg).

DMFP at doses of 0.1 and 0.5 mg/kg significantly increased response latency time at 150 min after the treatment in comparison to the control. The positive control group, morphine (5 mg/kg, i.p.), increased the response latency to heat stimulation 30 min after administration. This effect was maintained until 180 min after the treatment (*p* < 0.001).

#### 2.1.4. Capsaicin-Induced Paw-Licking Test

**Figure 5:**
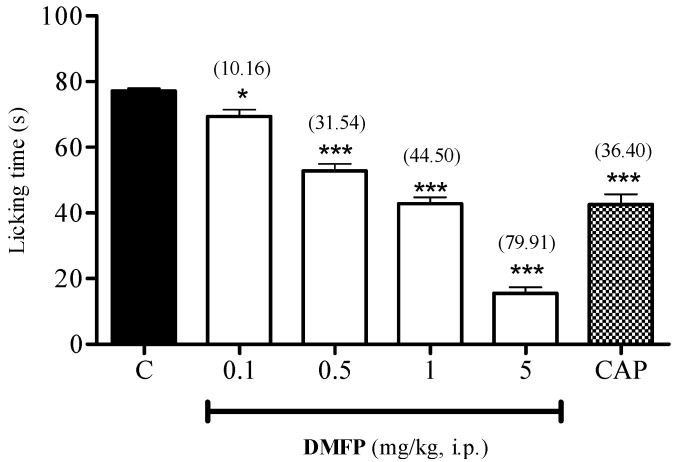
Effect of DMFP against capsaicin-induced paw-licking test in mice. Each column represents the mean ± S.E.M. of 6 mice. The mice were pre-treated with vehicle (C, 10 mL/kg, i.p.), DMFP (0.1, 0.5, 1.0 and 5 mg/kg, i.p.) or capsazepine (CAP, 0.17 mmol, i.p.) 30 min before intraplantar (i.pl.) injection of capsaicin (1.6 μg/paw, 20 μL). The asterisks denote the significance levels * *p* < 0.05, *** *p* < 0.001, when compared with control group (one-way ANOVA followed by Dunnett’s *post-hoc* test). Values in parentheses are percentage of inhibition.

The administration of DMFP produced significant dose-dependent inhibition of the capsaicin-induced neurogenic nociception at all doses, as compared to control group with a highest activity of 79.91% (*p* < 0.001) observed at the dose of 5.0 mg/kg (Figure 5). A similar inhibitory effect was also observed for capsazepine, which achieved 69.44% (*p* < 0.001) inhibition of capsaicin-induced nociception.

#### 2.1.5. Glutamate-Induced Paw-Licking Test

**Figure 6:**
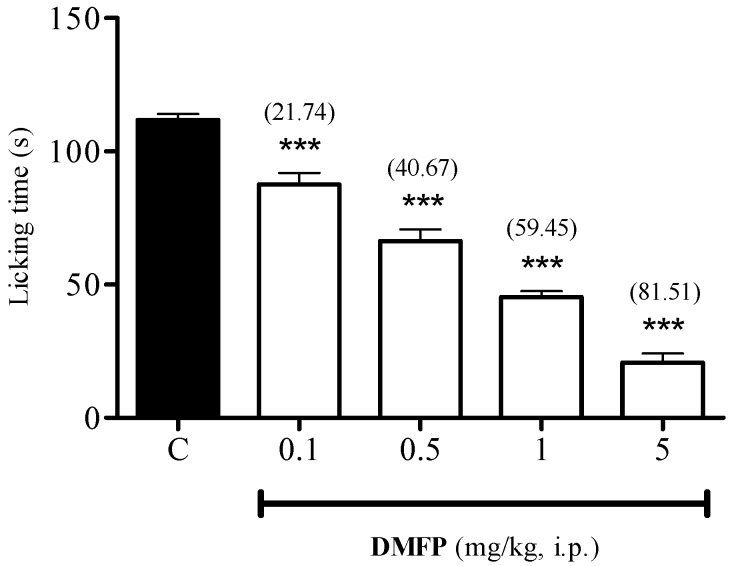
Effect of DMFP against glutamate-induced paw-licking test in mice. Each column represents the mean ± S.E.M. of 6 mice. The mice were pre-treated with vehicle (C), or DMFP (0.1, 0.5, 1 and 5 mg/kg, i.p.) 30 min before i.pl. injection of 20 μL of glutamate (10 μmol/paw). The asterisks denote the significance level *** *p* < 0.001, when compared with control group (one-way ANOVA followed by Dunnett’s *post-hoc* test). Values in parentheses are percentage of inhibition.

The results presented in Figure 6 show that the i.p. administration of DMFP at doses of 0.1, 0.5, 1.0 and 5.0 mg/kg caused significant dose-dependent inhibition of glutamate-induced nociception, with 21.74% (*p* < 0.001), 40.67% (*p* < 0.001), 59.45% (*p* < 0.001) and 81.51% (*p* < 0.001) of inhibition as compared to control, respectively.

#### 2.1.6. Involvement of Opioid Receptor

**Figure 7:**
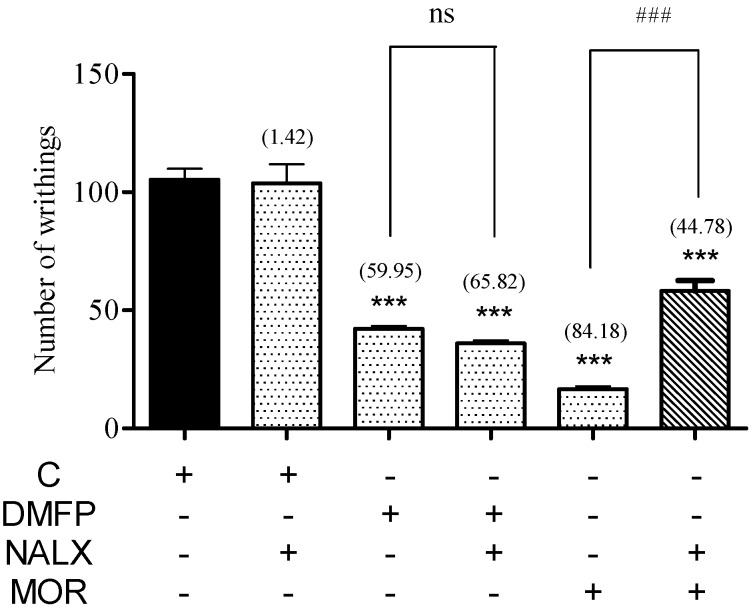
Effect of treatment with opioid antagonist, naloxone on the acetic acid-induced abdominal-writhing test in mice. Mice were pre-treated with naloxone (NALX, 5 mg/kg, i.p.) 15 min before the treatment with vehicle (C, 10 mL/kg, i.p), DMFP (0.1, 0.5, 1.0, 5.0 mg/kg, i.p.), or morphine (MOR, 5 mg/kg, i.p.). Each column represents the mean ± S.E.M. of six mice. The asterisks denote significance levels *** *p* < 0.001, when compared with vehicle control group; ### *p* < 0.001, when compared with morphine (MOR)-treated group; ns denotes no significance level when compared with DMFP-treated group (one-way ANOVA followed by Dunnett’s *post-hoc* test.)

**Figure 8:**
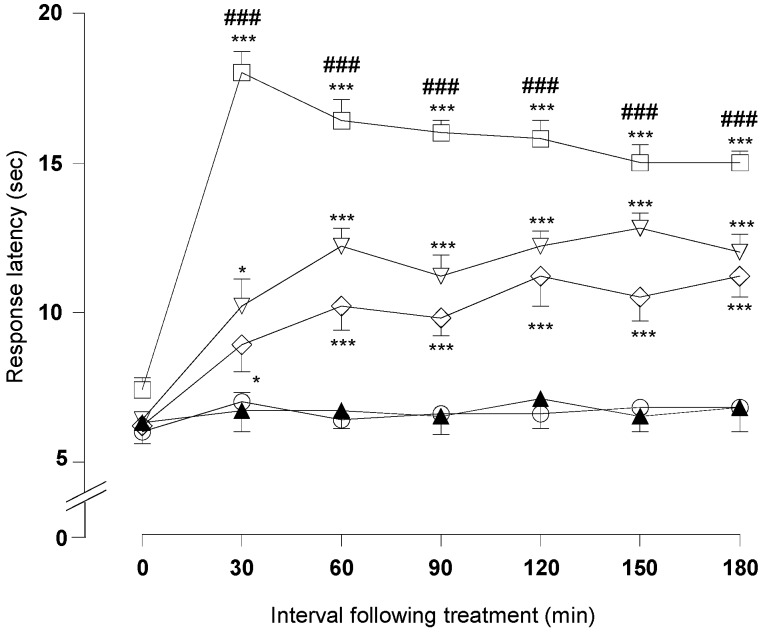
Effect of treatment with opioid antagonist, naloxone on the hot plate test in mice. Results are expressed in mean ± S.E.M. of response latency (s) of 6 mice. Statistical significance was denoted by one-way ANOVA followed by Dunnett’s *post-hoc* test. * *p* < 0.05; *** *p* < 0.001 compared with control group; ### *p* < 0.001, when compared with the group receiving appropriate drug/compound at the same dose without naloxone. Control (○), morphine (□, 5.0 mg/kg, i.p.), morphine + naloxone (▲, 5.0 + 5.0 mg/kg, i.p.), DMFP (▽, 5.0 mg/kg, i.p.) and DMFP + naloxone (◇, 5.0 + 5.0 mg/kg, i.p.).

The pre-administration of non-selective opioid receptor antagonist naloxone (5 mg/kg, i.p.) significantly antagonized the antinociceptive effect of morphine (5 mg/kg, i.p.) but was not able to antagonized the antinociceptive effect of DMFP (1 mg/kg, i.p.) in both the acetic acid-induced abdominal-writhing test (Figure 7) and the hot-plate test (Figure 8). Administration of naloxone per se did not affect both tests.

### 2.2. Motor Performance Study

#### Rota-Rod Test

**Figure 9:**
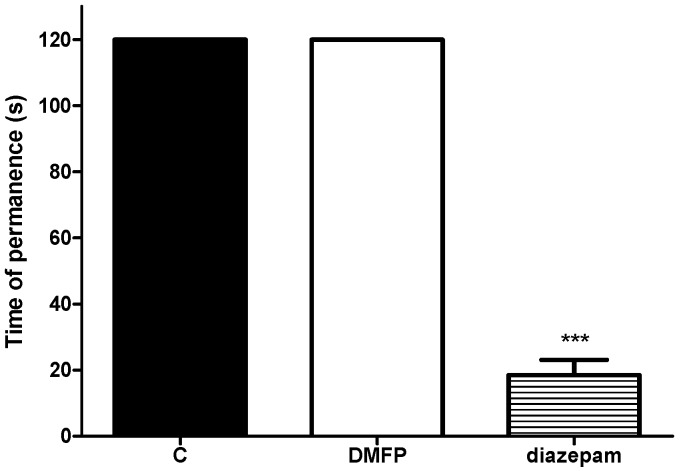
Effect of DMFP against on the Rota-rod test. Each column represents the mean ± S.E.M. in mice. Mice were pretreated with DMFP (5.0 mg/kg, i.p.) and diazepam (4.0 mg/kg, i.p.). Statistical analysis was determined by one-way ANOVA followed by Dunnett’s *post-hoc* test. *** *p* < 0.001 compared with control (C) group.

The administration of DMFP (5 mg/kg i.p.) did not cause any significant effect on the motor coordination of mice as assessed during the Rota-rod test when compared to the control group (Figure 9). In contrast, diazepam (4 mg/kg, i.p.) significantly reduced the time of permanence on the Rota-rod.

### 2.3. Toxicity Study

#### Preliminary Acute Toxicity Study

There were neither behavioral abnormalities nor mortality observed during 7 days period of observation in the treated mice. Gross morphological observation of stomachs and other organs of animals did not indicate any hemorrhagic lesions, ulcer or any other abnormalities.

## 3. Discussion

The present study established the antinociceptive effects produced by the intraperitoneal administration of a novel synthetic diarylpropanoid analogue, 3-(2,3-dimethoxyphenyl)-1-(5-methylfuran-2-yl)prop-2-en-1-one (DMFP) in mouse models of induced nociception and explored potential mechanisms of action. It was demonstrated for the first time that the administration of DMFP produced significant peripheral and central antinociceptive activity when tested in different in vivo chemicals and thermal experimental models of induced nociception, namely acetic acid-, formalin-, capsaicin-, glutamate-induced nociceptive tests and the hot-plate test.

The acetic acid-induced abdominal-writhing test is considered as a conventional animal model of pain and one of the most sensitive methods used for the screening of substances that have both analgesic origin and/or anti-inflammatory effects [21,22]. It was suggested that the introduction of acetic acid into the peritoneal cavity induces nociception by directly activating non-selective cationic channels located in the primary afferent pathways or indirectly by promoting the release of various endogenous algesic mediators such as prostaglandins, cytokines, bradykinin and others, as well as increasing lipoxygenase (LOX) and cyclooxygenase (COX) production in peripheral tissues [23,24,25,26]. The release of these endogenous substances with subsequent stimulation and sensitization of the peripheral primary afferent C-fibers neurons in the animal peritoneum produced a viscerosomatic reflex leading to abdominal constrictions [23,24,25,27]. The findings of the present study indicate that the administration (i.p.) of DMFP produced a significant dose-dependent reduction in the amount of abdominal writhing induced by acetic acid as compared to the control group. This finding suggests that the antinociceptive action of DMFP in the acetic acid-induced abdominal-writhing test could be the result of inhibited release of endogenous algesic mediators or direct inhibitory activity at nerve endings of the primary afferent neurons and/or inhibition of the transmission pathway entering the dorsal horn. 

Although the abdominal-writhing test has a very good sensitivity however it is non-specific in nature, as in some cases its writhing response can be suppressed by muscle relaxants and other types of drugs, which could lead to misinterpretation of the results. Furthermore, it does not exactly discriminate the involvement of a central or peripheral mechanism [19,22]. For these reasons, the possible inhibitory effect of DMFP was evaluated further in the formalin-induced paw-licking and hot-plate tests, which are more specific tests for the investigation of peripherally and centrally mediated antinociceptive activity [28,29].

The formalin-induced paw-licking test is a test model that produced two distinct phases of nociceptive responses. The first phase (neurogenic) which reflects centrally mediated pain, is evoked by direct formalin stimulation of the primary afferent C-fibers, result from the release of substance P, serotonin, kinins and/CGRP. In contrast, the second phase (inflammatory), which reflects peripherally mediated pain, is mainly due to either a subsequent inflammation reaction in the peripheral tissue mediated by the release of various inflammatory mediators such as prostaglandins, COX and NO, the effect of sensitizing dorsal horn neurons of the spinal cord or a combination of both [29,30]. The biphasic nature of the pain response in this test, which reflects different pathological processes, can be used to elucidate the mechanism involved in analgesia [29]. In addition, it is well appreciated that centrally acting drugs, such as opioids, inhibit both phases of pain, while peripherally acting drugs such as acetylsalicylic acid and dexamethasone that inhibit COX activity actually inhibit only the second phase [18,28,30]. The results of the present study show that the i.p. administration of DMFP significantly and dose-dependently attenuated the nociceptive response in both neurogenic and inflammatory phases of the formalin-induced paw-licking test in mice. Correspondingly, both phases of nociceptive response induced by formalin in the present study was also inhibited by the centrally acting drug, morphine, while the peripherally acting drugs, ASA, only inhibited the second phase of the test.

Similarly, the central antinociceptive effect of DMFP strongly supports the results obtained in the hot-plate test, which is a preferential method to screen centrally acting analgesic drugs. This effect is thought to demonstrate the involvement of central mechanisms (supraspinally) [22,31,32]. It was demonstrated that i.p. administration of DMFP exerts significant prolongation in the latency response time to heat stimuli of the hot-plate test. Taking into account the inhibitory property of the DMFP in the acetic acid-induced abdominal-writhing test, both phases of the formalin-induced paw-licking test and the hot-plate test, it is strongly suggested that DMFP acting both centrally and peripherally, which also implies that it possesses not only antinociceptive but also anti-inflammatory activity. As far as central analgesic effect of DMFP is concerned, however, other studies by means of intracerebroventricular and/or intrathecal routes of administration of DMFP are necessary to further substantiate the involvement of central nervous system in the central analgesic effect of DMFP. Another limitation of the present study is the lack of information on blood brain barrier (BBB) permeability of DMFP. Several studies have reported the ability of chalcones and its analogues to cross the BBB and have observed that it is possible for some chalcones to do so due to their lipophilic nature and their specific interactions with the efflux transporters expressed in the epithelial cells that make up the BBB [33,34] but for DMFP, its ability to cross the BBB is yet to determine. This information is crucial in order to determine the potential direct drug-protein interaction of DMFP with different types of receptors at the spinal or supraspinal levels cord, thus ascertain the exact mechanism of its central analgesic effect.

Numerous reports have indicated the involvement of various endogenous systems in pain control [25,35,36]. Therefore, in the present study we conducted preliminary experiments to explore the involvement of opioids, TRVP1 and glutamate receptors in DMFP-induced antinociception. The possible involvement of opioid receptors in the DMFP-induced antinociception was explored in a separate group of mice using the acetic acid-induced abdominal-writhing test and the hot-plate test, since the opioid receptors on peripheral terminals of primary afferent fibers can be the sites of the intrinsic modulation of nociception [37]. In addition, it was reported that drugs with action on these receptors may exerted analgesic effects without the presence of the central adverse effects caused by opioids [38,39]. Numerous studies have shown that naloxone is capable of antagonizing the opioid receptors in a non-selective manner [27,40]. Our findings showed that the inhibitory effect of DMFP in both test models, in contrast to that of morphine, was not antagonized by pre-administration with the non-selective opioid receptor antagonist, naloxone. The lacks of response to naloxone exclude the involvement opioid receptors in antinociceptive of DMFP. Thus, these results imply that both the peripheral and central mechanisms of DMFP-induced antinociception were neither mediated through the activation of opioid receptors nor the modulation of the effect of endogenous opioid peptides. 

Notably, DMFP significantly inhibited the nociceptive effect evoked by capsaicin and glutamate. Capsaicin is a pungent ingredient of red chili peppers that acts directly to activate vanilloid receptor type-1 (transient receptor potential cation channel V1 or TRPV-1) localized on nociceptive afferent C-fibers, in the dorsal root ganglion of the spinal cord, trigeminal ganglia and CNS [36,41,42]. The stimulation of TRPV1 receptors promotes vascular leakage and vasodilatation, culminating in the production and release of various neurotransmitters, such as glutamate and substance P, NO and pro-inflammatory mediators from the peripheral and central terminals of primary afferent neurons that contribute to nociceptive signal transmission and processing [41,42]. The results of the present study show that DMFP attenuates the neurogenic pain perception caused by capsaicin in a dose-dependent fashion. Also, it was observed that capsazepine, a selective capsaicin receptor antagonist, significantly antagonized the nociception induced by capsaicin. Thus, these findings suggest that DMFP possibly exerts its analgesic effect via inhibition of the nociceptive transmission initiated by TRPV1 receptors activation.

It is well appreciated that glutamate, a major excitatory amino acid neurotransmitter, participates in the processes involved in nociceptive transmission, development and maintenance of the nociceptive response through the activation of both ionotropic (iGluRs) and metabotropic glutamate receptors (mGluRs), leading to the excitation and sensitization of peripheral, central spinal and/or supraspinal nociceptors [43,44,45]. The iGluRs are nonselective ligand gated cation channels responsible for fast synaptic transmission eliciting currents of Ca^2+^, Na^+^ or K^+^, whereas the mGluRs belong to the large family of G-protein coupled receptors and are responsible for the neuromodulatory activity of glutamate through the formation of second messengers, mainly inositol 1,4,5-triphosphate, diacylglycerol and cyclic nucleotides. These receptors in different states and on different scales, participate in induction, modulation or maintenance of pain [46,47]. Indeed, drugs capable of inhibiting either iGluRs or/and mGluRs exhibit antinociceptive effects [45,48]. It was demonstrated that i.pl. injection of glutamate into the mouse hind paw results in an increased influx of calcium with the activation of neuronal NO synthase and NO formation [49]. The increase in the amount of NO subsequently increases the release of pro-inflammatory mediators such as cytokines, reactive oxygen species (ROS) and prostanoids and activates the formation of cyclic GMP, which enhances the inflammatory reaction and producing nociceptive behaviors of rapid onset and short duration [49,50,51]. Consequently, inhibition of these receptors or decreased glutamate release at the level of peripheral, spinal and/or supraspinal would lead to a decrease in pain perception. It was demonstrated in the present study that the administration of DMFP produces significant dose-dependent inhibition of the nociceptive response exerted by the i.pl. injection of glutamate in mice. Hence, the present results strongly suggest that, at least in part, the antinociceptive activity induced by DMFP during the glutamate test could be due to its interaction with the glutamatergic system and/or its ability to inhibit NO production. However, this hypothesis should be confirmed by other experiments on the mechanism of action of DMFP, which are currently in progress in our laboratory. This result also substantiates the results observed in formalin- and capsaicin-induced paw-licking tests, as both studies show that there is an increased level of glutamate as well as NO due to the stimulation of afferent C-fibers following injection of formalin or capsaicin into the paw [22,30,42].

It is a matter of great concern in the investigation of analgesic action of compounds if pharmacological administration of compounds causes other behavioral alterations, such as motor incoordination and sedation, which might be misinterpreted as analgesic activity. Therefore in order to avoid such misinterpretation, a behavioral evaluation was conducted to determine if DMFP-induced antinociception were influenced by any disturbances on the central nervous system using the Rota-rod test. It was observed that treatments with DMFP did not affect the motor activity of mice, thus exclude possible false-positive results in analgesia or the influence of possible non-specific effects, such as sedation or motor dysfunction. This result indicated that the behavioral responses observed in all nociception induced experimental models in the current study were not due to behavioral alterations, but was rather reflected a particular analgesic potential of DMFP.

Finally, to confirm the safety profiles of DMFP, our preliminary acute toxicological analysis was performed in animals that received single oral dose treatment of DMFP. It was demonstrated after 7 days of observation, the animals even treated with the highest dose of DMFP up to 1 g/kg, did not present any alteration in overall behavior and there was no occurrence of mortality. There were also no changes in the gross macroscopic observations of the stomach and other vital organs, such the heart, lung, liver, kidney and spleen. These results indicate that treatment with DMFP does not impair organ function as assessed in this study, thus presenting good safety conditions and suggesting the efficacy of the tested compound.

## 4. Materials and Methods

### 4.1. General Information

All the reagents and solvents used were purchased from Sigma-Aldrich Co. (St. Louis, MO, USA) and were used without further purification. The melting points (mp.) were determined on a hot-stage melting point apparatus, XSP-12 500X (Bibby Scientific Limited, Staffordshire, UK), and are uncorrected. IR spectra were recorded on a Perkin–Elmer RXI Fourier transform (FT) IR spectrometer (Perkin Elmer, Waltham, MA, USA) as KBr disks. Mass spectra were measured on a Finnigan Mat SSQ 710 spectrometer (Finnigan MAT, San Jose, CA, USA) with ionization induced by electron impact at 70 eV. Then ^1^H-NMR spectra were recorded in CDCl_3_ using a Varian 500-MHz NMR spectrometer (Varian Associates, Palo Alto, CA, USA). Column chromatography was performed on silica gel 60 Merck 9385 (230–400 mesh, ASTM). Thin layer chromatography (TLC) was performed on pre-coated silica plates (Merck Kiesegel 60F254, 0.2 mm thickness) sheets.

### 4.2. Synthesis of 3-(2,3-Dimethoxyphenyl)-1-(5-methylfuran-2-yl)prop-2-en-1-one (***DMFP***)

The synthetic diarylpropanoid analogue, DMPF (Figure 1) was chemically synthesized at the Institute of Bioscience, Universiti Putra Malaysia. Briefly, to a mixture of 2-acetyl-5-methylfuran (1.0 mmol) in ethanol (15 mL) was added with NaOH (1.5 mmol, 40%) and stirred for 10 minute in cold water. Then, added substituted 2,5-dimethoxybenzaldehyde (1.0 mmol) and stirred the reaction mixture at room temperature for 24 h. The progress of reaction was monitored by TLC and the reaction mixture was poured over crushed ice and acidified with acetic acid. The crude products were dissolved in distilled water and extracted with ethyl acetate. The yellow layer of EA was washed with water and dried over sodium sulfate anhydrous. The compound was purified by column chromatography using silica gel mesh size (100–200 mesh, Merck) and elution with petroleum ether and ethyl acetate. Yield: 82%; yellow crystals; m.p. 132–134 °C. IR(CHCl_3_) υ: 2937 (C–H stretch), 1652 (C=O), 1600 (C=C), 1513 (C=C), 1269 (C–O aromatic), 1074, 1001 cm^−1^; ^1^H-NMR (500 MHz, CDCl_3_): δ 2.44 (s, 3H, CH_3_), 3.89 (s, 6H, 2 × OCH_3_), 6.22 (d, *J* = 3.0 Hz, 1H, H-4 furanyl), 6.97 (d, *J* = 8.0 Hz, 1H, H-4 phenyl), 7.11 (t, *J* = 8.0 Hz each, 1H, H-5 phenyl), 7.28 (d, *J* = 8.0 Hz, 1H, H-6 phenyl), 7.46 (d, *J* = 16 Hz, 1H, H-α), 7.24 (d, *J* = 3.0 Hz, 1H, H-3 furanyl), 8.15 (d, *J* = 16 Hz, 1H, H-β). EIMS *m*/*z* (rel. int.) calculated for C_16_H_16_O_4_ (M^+^, %): 272 [M^+^].

### 4.3. Animals

Adult male ICR mice weighing 20–30 g were used. All mice were housed and maintained in the Animal Unit, Faculty of Medicine and Health Sciences, Universiti Putra Malaysia on a 12 h light/dark cycle with standard commercialized rodent diet and water available ad libitum. Experimental groups consisted of six randomly selected mice per group (*n* = 6), acclimatized for at least 1 h prior to the experiment and used only once throughout the experiments. All animal care and experimental protocols conducted in this study were approved by the Ethical Committee, Faculty of Medicine and Health Sciences, Universiti Putra Malaysia (ACUC_UPM⁄ FPSK⁄ PADS ⁄BR-UUH⁄00330). The number of animals and the intensities of noxious stimuli in the experimental protocols used were the minimum, just the amounts necessary to demonstrate consistent effects of the drug treatments. The animals used were euthanized by cervical dislocation immediately after completion of the experiment. In all experiments, data were collected in a blinded, randomized and controlled design.

### 4.4. Drugs and Chemicals

The following drugs and chemicals were used: morphine hydrochloride, acetylsalicylic acid (ASA), diazepam, Tween 20, absolute ethanol (100%), acetic acid, formalin, capsaicin, capsazepin, glutamate and naloxone hydrochloride (Sigma-Aldrich Co.). DMPF and ASA were dissolved in a vehicle containing absolute ethanol (5%), Tween 20 (5%) and normal saline (0.9% NaCl, 90%). The final concentrations of ethanol and Tween 20 did not exceed 10% and did not cause any effect per se. All other drugs and chemicals used were dissolved in normal saline. Respective controls received only vehicle. All drugs and DMPF solutions were prepared just before the experiments and administered via the intraperitoneal route (i.p.) at a volume of 10 mL/kg. Doses and drugs administration schedules were selected based our previous studies and on pilot experiments in our laboratory [9,13,18,19].

### 4.5. Antinociceptive Study

#### 4.5.1 Acetic Acid-Induced Writhing Test

The acetic acid-induced abdominal-constriction test was performed as described previously with slight modifications [19]. Experimental groups of mice were treated intraperitoneally (i.p.) with vehicle (10 mL/kg), DMFP (0.1, 0.5, 1.0, 5.0 mg/kg) or ASA (100 mg/kg) 30 min before the administration of acetic acid solution (0.6% *v*/*v*, 10 mL/kg, i.p.). Subsequently, the frequency of abdominal constriction in the mice was cumulatively counted for each mouse over a period of 30 min. Antinociceptive activity was expressed as a reduction in the number of abdominal constrictions between the control animals and the mice that were pre-treated with the compounds.

#### 4.5.2. Formalin-Induced Paw-Licking Test

The test was carried out as described previously [28], with minor modifications and licking of the formalin injected paw behavior was selected over flinching, shaking, lifting or favoring behavior as this was the established and optimized formalin-induced pain method used in our laboratory [9,19,35]. Briefly, 20 µL of 2.5% formalin solution (0.92% formaldehyde) in 0.9% saline solution was injected intraplantar (i.pl.) into the right hind paw of the mouse with a 27-gauge needle fitted to a microsyringe. The animal was then immediately placed into an observation perspex chamber and the amount of time spent licking the injected paw was considered as indication of pain. Two distinct phases of intensive licking and biting the paw activity were recorded using a chronometer during the first 5 min and 15–30 min, following formalin injection, and were measured as an early acute (neurogenic) phase and a late tonic (inflammatory) phase of pain response, respectively. The mice were i.p. treated with vehicle (10 mg/kg), DMFP (0.1, 0.5, 1.0, 5.0 mg/kg), ASA (100 mg/kg) or morphine (5 mg/kg), 30 min before formalin injection.

#### 4.5.3. Hot-Plate Test.

The hot-plate test was conducted to measure response latencies, according to a previously described method with slight modifications [9]. In this experiment, the mice were treated with vehicle (10 mL/kg, i.p.), DMFP (0.1, 0.5, 1.0, 5.0 mg/kg) or morphine (5 mg/kg, i.p.) and placed individually on a hot plate (Model 7280, Ugo Basile, Varese, Italy) maintained at 52 ± 0.5 °C. All substances were administered 30 min before the beginning of the experiment. The time elapsed between placement of the animal on the heated surface of the hot plate and the occurrence of either licking of the hind paws, shaking the paws or jumping off the surface was recorded as response latency. The response latency was observed just before treatment (time 0) and 30, 60, 90, 120, 150 and 180 min after substance administration. A latency of 20 s was defined as complete analgesia and used as a cut-off time to avoid tissue injury to the animal’s paws. Mice in this experiment were selected 24 h before the experiment, and only those showing response latency within the range of 5–8 s were used for the experiment. 

#### 4.5.4. Capsaicin-Induced Paw-Licking Test

The method performed was similar to that described previously with slight modification [42,52]. Mice received an intraplantar injection (i.pl.) of capsaicin (20 μL, 1.6 μg/paw) in the plantar surface of the right hind paw. Immediately after the capsaicin injection, the animals were placed individually in an observation perspex box and the amount of time the animal spent licking or biting the capsaicin-injected paw was recorded for a period of 5 min and was considered as the nociceptive response. The animals were pre-treated with vehicle (10 mL/kg, i.p.) or DMFP (0.1, 0.5, 1.0, 5.0 mg/kg, i.p.) 30 min before injection of capsaicin. In separate experiments animals received an intraplantar injection of capsazepine (a capsaicin receptor antagonist, 0.17 mmol/kg, i.p.) 30 min before the capsaicin injection.

#### 4.5.5. Glutamate-Induced Paw-Licking Test

The method used for glutamate-induced paw licking was similar to that described previously [49]. Mice were intraperitoneally pre-treated with vehicle (10 mL/kg, i.p.) or DMFP (0.1, 0.5, 1.0, 5.0 mg/kg, i.p.) 30 min before the i.pl. administration of glutamate (10 μmol/paw, 20 μL) in the ventral surface of the right hindpaw. Immediately, animals were individually placed in a transparent perspex box and the time that the animal spent licking or biting the glutamate-injected paw was recorded for a period of 15 min and was considered as indication of nociception.

#### 4.5.6. Involvement of Opioid Receptors

The possible participation of the opioid system in DMFP antinociceptive effect was evaluated in separate groups of animals using the acetic acid-induced abdominal writhing and the hot-plate tests. Mice were pre-treated with naloxone (5 mg/kg, i.p.) 15 min before the treatment with vehicle (10 mL/kg, i.p), DMFP (0.1, 0.5, 1.0, 5.0 mg/kg, i.p.), ASA (100 mg/kg, i.p.) or morphine (5 mg/kg, i.p.). 30 min after the treatments, the animals were subjected to the acetic acid-induced abdominal writhing and hot-plate tests as described previously [9,18].

### 4.6. Motor Performance Study

#### Rota-Rod Test

The Rota-rod test was conducted to investigate whether DMFP has non-specific muscle relaxant or sedative effects [53]. The Rota-rod apparatus (Model 7600, Ugo Basile) consisted of a bar with diameter of 3 cm, and the rotating speed was set at speed of 20 rpm. The latency for the mice to fall from the rotating rod were recorded. The animals were selected 24 h previously by eliminating those which did not remain on the bar for two consecutive periods of 60s. The mice were given treatment with vehicle (10 mg/mL, i.p.), DMFP (5 mg/kg, i.p.), or diazepam (4 mg/kg, i.p.) 30 min before being tested. Motor performance was evaluated at 0, 30 and 60 min following the treatment. Each mouse was subjected to two consecutive tests and results were averaged and expressed as the time (s) that the animal remained on the rotated bar of the Rota-rod. The cut-off time was 60 s.

### 4.7. Toxicity Study

#### Preliminary Acute Toxicity Test

The acute toxicity test was carried out to evaluate a possible acute toxicity effect of DMFP as previously described with some modifications [54]. Groups of mice received a single oral dose (p.o.) of vehicle (10 mL/kg) or DMFP (1 g/kg) during 7 days period and their behavioral parameters (i.e., convulsion, constipation, sedation, respiration, food and water intake) or mortality were observed. On day 8 of observations, the animals were euthanized; organs were removed and opened to observe any abnormalities (i.e., hyperemia and ulcer).

### 4.8. Statistical Analysis

Experimental groups consisted of six mice or rats. Data are presented as means ± S.E.M. The data were analyzed by one-way analysis of variance (ANOVA) with Dunnett’s test for *post-hoc* comparisons of different treatment groups with vehicle control. A value of *p* < 0.05 was considered statistically significant in all tests. The effective dose 50 (ED_50_; dose producing a 50% reduction in the total number of abdominal writhes) and 95% confidence interval (CI) values in the acetic acid-induced abdominal-constriction test were determined by linear regression using GraphPad 5 (GraphPad Software Inc., San Diego, CA, USA).

## 5. Conclusions

In conclusion, we have demonstrated, through several in vivo experiments of induced nociception in mice, that the administration of a novel synthetic diarylpropanoid analogue (DMFP), at a dose that did not cause any toxic effects or interfere with motor coordination, produced prominent peripheral and central antinociceptive effects. Based on the current results, DMFP’s peripheral antinociceptive activity appears to be associated with the attenuated production and/or release of NO and various pro-inflammatory mediators, while central antinociceptive activity seems to be unrelated to the opioidergic system but could involve, at least in part, an interaction with the inhibition of capsaicin-sensitive fibers and the glutamatergic system. The present observations on the numerous targets involved contribute to a better understanding of the peripheral and central antinociceptive mechanisms of action induced by DMFP, which also offer a promising option for further investigation as an effective treatment for visceral and inflammatory pain.
